# *Diospyros kaki* and *Citrus unshiu* Mixture Improves Disorders of Lipid Metabolism in Nonalcoholic Fatty Liver Disease

**DOI:** 10.1155/2020/8812634

**Published:** 2020-12-24

**Authors:** Mi-Rae Shin, Sung Ho Shin, Seong-Soo Roh

**Affiliations:** ^1^Department of Herbology, Korean Medicine of College, Daegu Haany University, Daegu 42158, Republic of Korea; ^2^Foot-and-Mouth Disease Vaccine Research Center, Animal and Plant Quarantine Agency, Gyeongsangbuk-do 39660, Republic of Korea

## Abstract

Nonalcoholic fatty liver disease (NAFLD) has been a major cause of a chronic liver disease over recent decades and increasing worldwide in parallel with the remarkable growth of obesity. In the present study, we investigate the ameliorative effects of PCM, a combination of *Diospyros kaki* fruit and *Citrus unshiu* peel mixture, on high-fat diet- (HFD-) induced NAFLD and clarify the potential mechanisms. PCM in HFD-fed mice was orally administered at a dose of 50 or 100 mg/kg subsequently for 2 months. Thereafter, lipid metabolism parameters and fat synthesis-related genes in the mouse liver were evaluated. Subsequently, body weight changes, liver weight, serum liver function and lipid profiles, and liver pathology were examined, and the relative levels of fatty acid synthesis and *β*-oxidation gene expression were evaluated by western blot. Serum AST, ALT, and TG levels in the HFD control mice were significantly higher than those of normal mice. Compared with HFD control mice, PCM supplementation increased phosphorylation of AMP-activated protein kinase (AMPK). Peroxisome proliferator-activated receptor (PPAR) *α* was significantly increased by PCM administration. Continuously, the activation of PPAR*α* significantly elevated carnitine palmitoyltransferase 1 (CPT-1), a key enzyme in fatty acid *β*-oxidation, and mitochondrial uncoupling protein 2 (UCP-2), thermogenic regulatory genes, in PCM-treated mice compared with those of HFD control mice. Moreover, PCM inhibits lipogenesis and cholesterol synthesis via suppression of sterol regulatory element binding protein-1 (SREBP-1) and SREBP-2 and its target genes such as acetyl-CoA carboxylase (ACC), fatty acid synthase (FAS), stearoyl-CoA desaturase-1 (SCD-1), and 3-hydroxy-3-methylglutaryl coenzyme A reductase (HMGCR). Taken together, these effects were mediated through activation of AMPK. In the conclusion, PCM improved liver damage in HFD-fed mice and attenuated NAFLD by the activation of PPAR*α* and the inhibition of SREBPs expression via AMPK-dependent pathways.

## 1. Introduction

Nonalcoholic fatty liver disease (NAFLD) is the most common etiology of chronic liver disease and characterized by the accumulation of triglycerides in liver hepatocytes of patients without the excess consumption quantities of alcohol. The prevalence of NAFLD is constantly rising worldwide in parallel with overnutrition associated with high-fat and high-carbohydrate intake [1]. NAFLD patients are commonly accompanied with obesity, diabetes, and hyperlipidemia, but not all obese people develop into NAFLD, and the various factors may play the important role in the pathogenesis of NAFLD. Especially, its worldwide incidence ranges from 9 to 37% [2], while the current prevalence of patients with the nonobese and nondiabetic status in the Asia-Pacific region was from 15 to 21% and has been increasing with time [3]. Accordingly, there is a general consensus that body control with lifestyle modification is the basic and effective treatment for NAFLD, although insulin sensitizers, such as metformin and thiazolidinedione, and antioxidants have some beneficial and therapeutic effects [4]. Therefore, a lot of attention has been focused on natural products, which increase fat oxidation and decrease adipogenesis and regulate lipid metabolism effectively.

According to the previous study, the immoderate intake of fat altered mitochondrial lipid composition, which can affect its function [5, 6]. Dietary acetic acid is metabolized to acetyl-CoA with the production of AMP. The AMP-activated protein kinase (AMPK), an energy sensor, is activated by increase in the cellular AMP:ATP ratio caused by multiple stresses and induces metabolic changes through the modulation of gene expressions concerned in metabolic regulation [7]. Namely, AMPK maintains cellular energy homeostasis through the inhibition of fatty acid synthesis and the activation of fatty acid oxidation [8]. In this context, many researchers reported that these resulted from the inhibition of the cleavage and transcriptional activation of SREBP via direct phosphorylation of AMPK in the liver [6, 9]. Thus, AMPK has been recognized as a significant target for the management of obesity and hyperlipidemia.

PCM is an herbal formulation of *Diospyros kaki* fruit (Young persimmon) and *Citrus unshiu* peel. Both of these herbs are the most well-known traditional herbal medicines, frequently used to treat obesity [10]. The major bioactive components in *Diospyros kaki* fruit are nine specific carotenoids (i.e., *α*-carotene, *β*-carotene, lycopene, lutein, zeaxanthin, neoxanthin, violaxanthin, 9-cis-violaxanthin, and *β*-cryptoxanthin) [11] and polyphenols including various tannins (i.e., low molecular tannins—proanthocyanidins [12]—and highly polymerized tannins—tannins composed of epicatechin, epigallocatechin, epicatechin-3-O-gallate, and epigallocatechin-3-O-gallate [13]). The major groups of bioactive components in *Citrus unshiu* peel are flavonoids, carotenoids, and phenethylamine alkaloids [14]. The biological and multiple compounds of two herbs, *Diospyros kaki* fruit and *Citrus unshiu* peel, exerted pharmacological effects such as hypolipidemic [15–17], antioxidant [18], antiaging [12], antimicrobial, and antiviral activities [19]. Moreover, we reported that *Diospyros kaki* fruit and *Citrus unshiu* peel mixture inhibited triglyceride absorption via the inhibition of pancreatic lipase as in the previous study [20].

However, the ameliorative effect of *Diospyros kaki* fruit and *Citrus unshiu* peel extracts via regulation of lipid metabolism in an NAFLD model has not been investigated. To provide mechanistic explanation, we also investigated the role of AMPK protein and its downstream genes.

## 2. Materials and Methods

### 2.1. Chemicals and Reagents

The protease inhibitor mixture ethylenediaminetetraacetic acid (EDTA) was purchased from Wako Pure Chemical Industries, Ltd. (Osaka, Japan). 2′,7′-Dichlorofluorescein diacetate (DCF-DA) was obtained from Molecular Probes (Eugene, OR, USA). Rabbit polyclonal antibodies against peroxisome proliferator-activated receptor *α* (PPAR*α*), sterol regulatory element binding protein-1 (SREBP-1) and sterol regulatory element binding protein-2 (SREBP-2), 3-hydroxy-3-methylglutaryl coenzyme A reductase (HMGCR), and mouse monoclonal antibodies against fatty acid synthase (FAS), *β*-actin, and histone, and goat polyclonal antibody against mitochondrial uncoupling protein 2 (UCP-2) and stearoyl-CoA desaturase-1 (SCD-1) were purchased from Santa Cruz Biotechnology, Inc. (Santa Cruz, CA, USA). Rabbit polyclonal antibodies against acetyl-CoA carboxylase (ACC), AMPK*α*, and phosphor-AMPK*α* were purchased from Cell Signaling Technology, Inc. (Danvers, MA, USA). ECL Western Blotting Detection Reagents and nitrocellulose membranes were supplied by GE Healthcare (Buckinghamshire, UK).

### 2.2. Preparation of *Diospyros kaki* and *Citrus unshiu* Mixture

An unripe *Diospyros kaki* fruit was harvested in Gyeongsangbuk-do Agricultural Research & Extension Services (Sangju, Korea), and a dried *Citrus unshiu* peel was purchased from MSC Co., Ltd. (Yangsan, Korea). An unripe Diospyros kaki fruit (without removing the peel) was used raw, and only dried peel of Citrus unshiu was used. 500 kg of each was prepared and extracted with 5 times of water and boiled in 100°C for 2 h. Then, enzyme decomposition was carried out for 15 h. Next, the enzyme was inactivated at 90°C for 30 min. After filtration using diatomite, the extracts were concentrated till 30 Brix. The concentrated extracts were added with dextrin (15 calories/3.8 g carbohydrates in 4 g), which is used in powdered processed foods for the prevention from being condensed or hardened by moisture. Thereafter, they were sterilized at 95°C for 30 min. The sterilized extracts were freeze-dried and powered using a grinder. We obtained a yield of 21% by weight.

### 2.3. Experimental Animals and Treatment

The following information related to ethical approval (i.e., approving body and any reference numbers) was supplied: All animal experiments for the study were carried out following the Guide for the Care and Use of Laboratory Animals and were approved by the Animal Ethics Committee of Daegu Haany University (certificate number: DHU2015-010). Six-week-old ICR male mice (20–25 g) that are obese were purchased from Orient (Gyeonggi-do, Korea). Mice were maintained under a 12-h light/dark cycle and housed in a controlled environment (temperature 22 ± 3°C, humidity 55 ± 5%). The mice were allowed free access to laboratory pellet chow and water ad libitum. After adaptation (1 week), all experimental mice except normal mice were fed with 45% high-fat diet (Diet 12451; Research Diets, Inc., New Brunswick, NJ, USA) for 23 weeks freely without a special action until the starting point of drug treatment. Then, the body weight and food and water intake were determined every day during the drug treatment period (8 weeks). Male ICR mice (*n* = 35) were arbitrarily divided into five groups (*n* = 7 in each group): a normal group, HFD control group, orlistat group (60 mg/kg/day), and two PCM treatment groups (50 and 100 mg/kg/day). The normal and HFD control groups were provided water using a stomach tube, while the drug treatment groups were orally administered orlistat or PCM dissolved in water daily using a stomach tube for 8 weeks. After administration for 8 weeks, each mouse was etherized after fasting for 12 h. The serum was immediately separated from the blood by centrifugation. Subsequently, the liver was perfused through the artery with ice-cold physiological saline (0.9% NaCl, pH 7.4), removed, quickly frozen, and kept at −80°C until analysis.

### 2.4. Measurement of Hepatic Functional Parameters

Hepatic functional parameters (alanine aminotransferase (ALT) and aspartate aminotransferase (AST) assays were conducted spectrophotometrically using commercially available kits (Transaminase CII-Test; Wako Pure Chemical Industries, Ltd., Osaka, Japan).

### 2.5. Measurement of TG, TC, HDL, and LDL Contents in Serum and the Liver Tissue

The liver tissue was homogenized on ice with 0.9% cold NaCl buffer. Then, the homogenate was extracted with a mixture of chloroform and methanol (2 : 1, v/v) according to the method of Folch et al. [21]. The mixture was centrifuged at 1670 ×g for 15 min. The organic layer was collected and dried, and the residue was dissolved in isopropanol. Determination of triglyceride, total cholesterol, HDL, and LDL contents in serum and the liver tissue was performed using a commercial kit from Asan Pharm. Co., Ltd., (Cat. AM202, AM157S, AM203; Hwaseong-si, South Korea).

### 2.6. Preparation of Nuclear and Cytosol Fractions

Protein extraction was performed according to the method of Komatsu with minor modifications [22]. Esophageal tissues for cytosol fraction were homogenized with ice-cold lysis buffer A (250 mL) containing 10 mM HEPES (pH 7.8), 10 mM KCl, 2 mM MgCl_2_, 1 mM DTT, 0.1 mM EDTA, 0.1 mM PMSF, and 1,250 *μ*L protease inhibitor mixture solution. The homogenate incubated at 4°C for 20 min. Then, 10% NP-40 was added and mixed well. After centrifugation (13,400 ×g for 2 min at 4°C) using Eppendorf 5415R (Hamburg, Germany), the supernatant liquid (cytosol fraction) was separated using a new e-tube. The left pellets were washed twice with buffer A, and the supernatant was discarded. Next, the pellets were suspended with lysis buffer C (20 mL) containing 50 mM HEPES (pH 7.8), 50 mM KCl, 300 mM NaCl, 1 mM DTT, 0.1 mM EDTA, 0.1 mM PMSF, 1% (v/v) glycerol, and 100 *μ*L protease inhibitor mixture solution and incubated at 4°C for 30 min. After centrifugation (13,400 ×g for 10 min at 4°C), the nuclear fraction was prepared to collect the supernatant. Both cytosol and nuclear fractions were kept at −80°C before the analysis.

### 2.7. Western Blot Analyses

Postnuclear proteins for p-AMPK*α*, AMPK*α*, ACC, FAS, SCD-1, HMGCR, and *β*-actin and nuclear proteins for PPAR*α*, SBEBP-1, SREBP-2, and histone were electrophoresed in 8–13% sodium dodecyl sulfate polyacrylamide gel (SDS-PAGE). The separated proteins were transferred to a nitrocellulose membrane, blocked with 5% (w/v) skim milk solution for 1 h, and then incubated with primary and secondary antibodies for 1.5 h at room temperature. Each antigen-antibody complex was visualized using ECL Western Blotting Detection Reagents and detected by chemiluminescence with SENSI-Q2000 Chemidoc (Lugen Sci. Co., Ltd, Gyeonggi-do, Korea). Band densities were determined using ATTO Densitograph Software (ATTO Corporation, Tokyo, Japan) and quantified as the ratio to histone and *β*-actin. The protein levels of groups are expressed relative to those of normal mice.

### 2.8. Oil Red O Stain of the Liver Tissue

The microscopic analysis of the effect of PCM treatment on lipid accumulation of the HFD-fed mouse liver was carried out though Oil Red O stain. For Oil Red O staining, frozen liver tissue was cut into 7 *μ*m with ProbeOn Plus slides (Thermo Fisher Scientific) and affixed to microscope slides. Sections were reacted with Oil Red O solution buffer for 7 min at 60°C and then incubated with 85% propylene glycol for 3 min. After rinsing with water, sections were stained with Harris hematoxylin for counterstaining.

### 2.9. Hematoxylin and Eosin (H/E) Stain of the Adipose Tissue

For microscopic evaluation, the adipose tissue was cut to isolate the middle segment. This segment was fixed in 10% neutral-buffered formalin and, after embedding in paraffin, cut into 2 *μ*m sections and stained using hematoxylin and eosin (H/E) for microscopic evaluation. The stained slices were subsequently observed under an optical microscope and analyzed using the i-Solution Lite software program (InnerView Co.).

### 2.10. Statistical Analysis

Data were expressed as mean± SEM. Statistical comparisons were assessed by one-way ANOVA followed by an LSD test (SPSS 25.0 for Windows, SPSS Inc., USA), and *p*values < 0.05 were considered significant.

## 3. Results

### 3.1. Food and Water Intake, Body Weight Gain, and Liver Tissue Weight

Table 1 shows the food and water intake, body weight gain, and liver weight during the experimental periods. As shown in Table 1, normal mice started with significantly different mean body weights compared with HFD-fed mice (29.9 g and 43.3 g, respectively, *p* < 0.001). The increased liver weight at the end of experimental period (31 weeks) significantly decreased by the administration of O60 and PCM100 except PCM50 (without significance). Weight gain of normal mice increased slightly during the studied period of 31 weeks, but mice treated with O60 for 8 weeks had a significantly decreased weight gain, whereas PCM-treated mice for 8 weeks showed a tendency to decrease (without significance) weight gain. High- fat diet causes an increase in the weight of liver. Therefore, pretreatment with O60, PCM50, and PCM100 effectively prevented the increase of liver weight (23.2%, 7.2%, and 26.0% of HFD control value, respectively). Moreover, the food intake was not significantly different between HFD control mice and PCM-treated mice except O60-treated mice. Although food intake of O60-treated mice significantly increases, body weight gain significantly decreases. As a result, PCM treatment decreased the weight gain and liver weight. Herein, PCM may help to improve the disorders of HFD-induced NAFLD.

### 3.2. Biochemical Analyses

Table 2 shows that high-fat diets caused an increase in serum ROS, TG, TC, LDL, and VLDL (*p* < 0.001, *p* < 0.01, *p* < 0.001, *p* < 0.001, and *p* < 0.05, respectively). The augmented ROS, TG, and VLDL levels were significantly lowered by O60 or PCM compared with the HFD control mice. Moreover, the administration of O60 or PCM50 elevated significantly the HDL level (*p* < 0.01, *p* < 0.001), whereas PCM100 increased (without significance) the level slightly. Excess fat intake leads to immoderate lipid accumulation and ROS overproduction. Like this, HFD control mice displayed significantly increased ROS and TBARS levels in the liver, and in the drug-treated mice compared with HFD control mice, these levels lowered markedly. In addition, the levels of hepatic TG and TC significantly ameliorated by the administration of O60 or PCM100, but PCM50 reduced the TG level slightly.

### 3.3. Hepatic Functional Parameters

Table 3 shows the effects of PCM on serum hepatic functional parameters such as AST and ALT. HFD control mice showed significantly higher AST and ALT levels than normal mice, while the elevated AST level was significantly reduced after PCM administration (*p* < 0.01, *p* < 0.001, *p* < 0.001, respectively) and the ALT level was significantly reduced after PCM administration (*p* < 0.001, *p* < 0.001, *p* < 0.001, respectively).

### 3.4. Histological Changes

**Figure 1:**
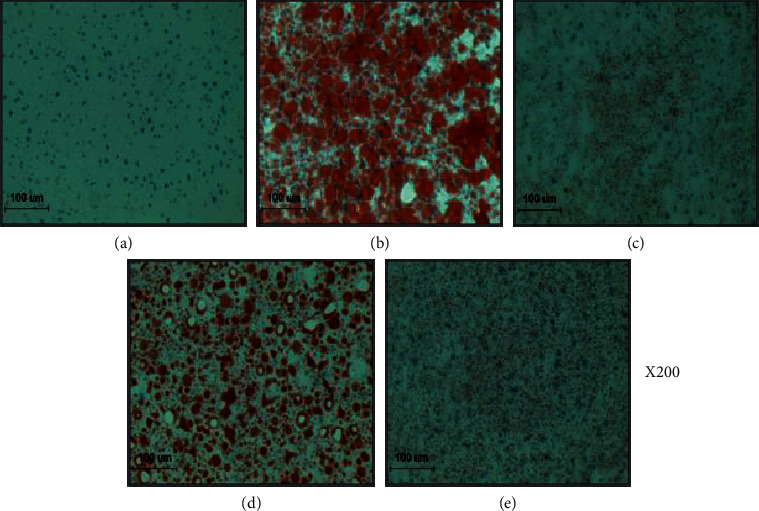
Oil red O staining of hepatic tissue: (a) normal mice, (b) HFD control mice, (c) orlistat 60 mg/kg-treated and obese mice, (d) PCM 50 mg/kg-treated and obese mice, and (e) PCM 100 mg/kg-treated and obese mice (×200).

**Figure 2:**
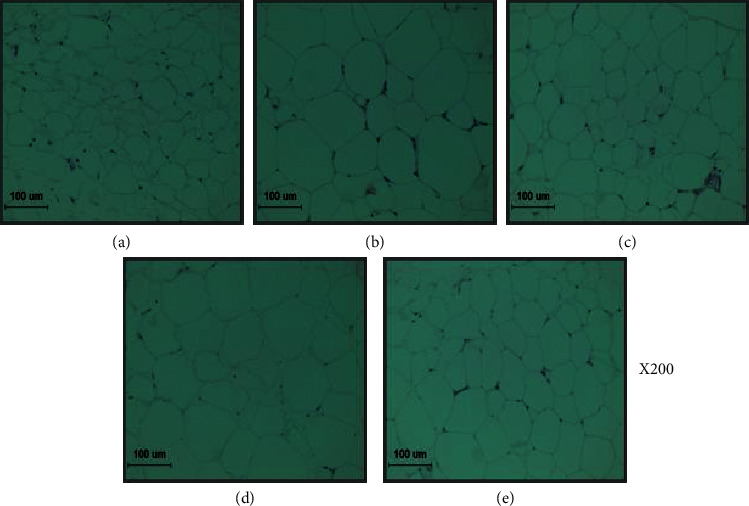
H/E staining of adipose tissue: (a) normal mice, (b) HFD control mice, (c) orlistat 60 mg/kg-treated and obese mice, (d) PCM 50 mg/kg-treated and obese mice, and (e) PCM 100 mg/kg-treated and obese mice (×200).

As shown in Figures 1 and 2, we investigate whether the improved lipid metabolism had changed at the size of adipocyte and lipid accumulation. We observed H&E in adipose tissue and Oil red O staining in the liver to evaluate the extent of lipid accumulation and fat size. As expected, the HFD-fed mouse liver resulted in severe hepatic lipid accumulation, characterized by an increase fat size, while O60 or PCM100 supplementation more effectively improved the pathological status compared with the HFD control mice.

### 3.5. AMPK Phosphorylation Protein Expression in the Liver

**Figure 3:**
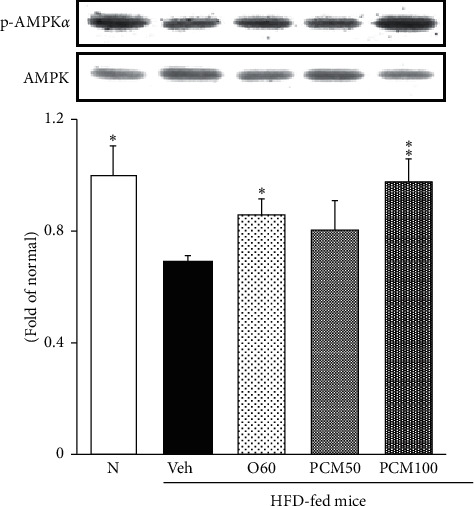
p-AMPK protein expressions in the liver. N, normal mice; Veh, HFD control mice; O60, orlistat 60 mg/kg-treated and obese mice; PCM50, PCM 50 mg/kg-treated and obese mice; PCM100, PCM 100 mg/kg-treated and obese mice. Data are the mean ± SEM, *n* = 7. Significance: ^*∗*^*p* < 0.05 and ^*∗∗*^*p* < 0.01 versus HFD control mice. p-AMPK, phosphor-AMP-activated protein kinase.

Figure 3 shows that protein expression of AMPK phosphorylation in the liver. p-AMPK*α* was lower in HFD control mice, but the administration of O60 and PCM for 8 weeks significantly elevated p-AMPK*α* in the liver (*p* < 0.05 and *p* < 0.01, respectively).

### 3.6. Fatty Acid Oxidation-Related Protein Expressions in the Liver

**Figure 4:**
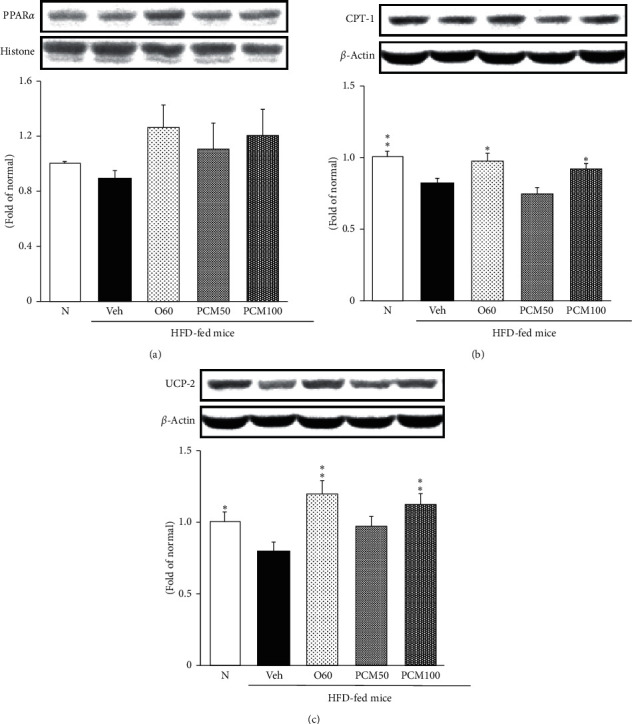
PPAR*α* (a), CPT-1 (b), and UCP-2 protein (c) expressions in the liver. N, normal mice; Veh, HFD control mice; O60, orlistat 60 mg/kg-treated and obese mice; PCM50, PCM 50 mg/kg-treated and obese mice; PCM100, PCM 100 mg/kg-treated and obese mice. Data are the mean ± SEM, *n* = 7. Significance: ^*∗*^*p* < 0.05 and ^*∗∗*^*p* < 0.01 versus HFD control mice. PPAR*α*, peroxisome proliferator-activated receptor *α*; CPT-1, carnitine palmitoyltransferase 1; UCP-2, uncoupling protein 2.

Disturbance of peripheral lipid storage in obesity may result in lipid overloading to the liver, leading to hepatic lipid accumulation in the form of triglycerides. Then, it tries to increase fatty acid oxidation in context of body homeostasis.  Figure 4 reveals that HFD-fed mice dramatically reduced the protein expressions of fatty acid oxidation markers (PPAR*α* and CPT-1). However, PCM treatment significantly reversed the decreased PPAR* α *level. Moreover, its target gene CPT-1 significantly increased compared with those of HFD control mice. Uncoupling protein 2 (UCP2) performs a biological role in the energy balance and thermogenesis. The result of the present study indicates that the decreased expression of UCP2 in HFD control mice was significantly increased by PCM treatment.

### 3.7. Transcription Genes Controlling TG and Cholesterol Synthesis in the Liver

**Figure 5:**
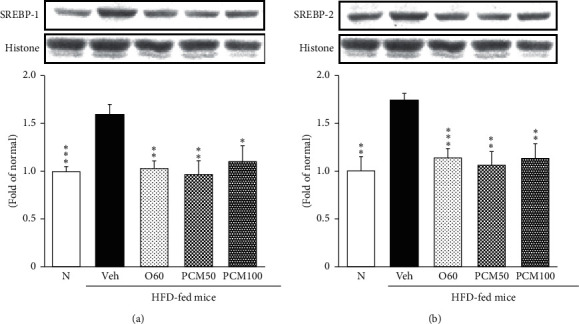
SREBP-1 (a) and SREBP-2 (b) protein expressions in the liver. N, normal mice; Veh, HFD control mice; O60, orlistat 60 mg/kg-treated and obese mice; PCM50, PCM 50 mg/kg-treated and obese mice; PCM100, PCM 100 mg/kg-treated and obese mice. Data are the mean ± SEM, *n* = 7. Significance: ^*∗*^*p* < 0.05, ^*∗∗*^*p* < 0.01, and ^*∗∗∗*^*p* < 0.001 versus HFD control mice. SREBP-1, sterol regulatory element binding protein-1; SREBP-2, sterol regulatory element binding protein-2.

As shown in Figure 5, SREBP-1 and SREBP-2 in HFD control mice were significantly increased compared with normal mice; however, SREBP-1 and SREBP-2 in PCM100-treated obese mice were significantly lower than that in HFD control mice. In addition, the levels of SREBP-1 and SREBP-2 by AMPK activation were nearly decreased to normal levels in O60 and PCM mice.

### 3.8. Protein Expression Led to TG and Cholesterol Synthesis in the Liver

**Figure 6:**
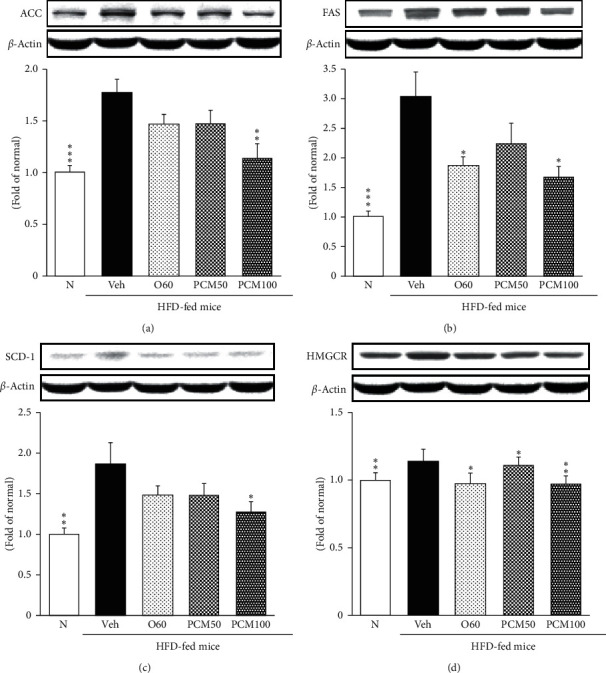
ACC (a), FAS (b), SCD-1 (c), and HMGCR (d) protein expressions in the liver. N, normal mice; Veh, HFD control mice; O60, orlistat 60 mg/kg-treated and obese mice; PCM50, PCM 50 mg/kg-treated and obese mice; PCM100, PCM 100 mg/kg-treated and obese mice. Data are the mean ± SEM, *n* = 7. Significance: ^*∗*^*p* < 0.05, ^*∗∗*^*p* < 0.01, and ^*∗∗∗*^*p* < 0.001 versus HFD control mice. ACC, acetyl-CoA carboxylase; FAS, fatty acid synthase; SCD-1, stearoyl-CoA desaturase-1; HMGCR, 3-hydroxy-3-methylglutaryl coenzyme A reductase.

ACC, FAS, and SCD-1, a target of SREBP-1, is associated with lipogenesis. The activation of SREBP-1 upregulated lipid metabolism-related proteins such as ACC [23]. This study showed that the level of ACC was significantly upregulated by HFD intake. Accordingly, the marked elevation of fatty acid synthesis markers such as ACC, FAS, and SCD-1 expressed in HFD control mice. However, PCM administration effectively decreased such markers. HMGCR protein expression was significantly elevated in HFD control mice compared with normal mice, whereas PCM-treated mice were significantly decreased. (Figure 6). HMGCR, a target of SREBP-2, is the rate-limiting enzyme of cholesterol synthesis and its decreased expression is significantly elevated by the O60 and PCM administration.

## 4. Discussion

Nonalcoholic fatty liver disease (NAFLD) is accompanied by several metabolic dysfunction and diseases such as obesity, type 2 diabetes mellitus, and dyslipidemia (increased serum triglyceride, cholesterol, or LDL and decreased HDL). Obesity and metabolic syndrome are due to excess energy intake and nutrient availability. Energy restriction and appropriate exercise and selective drug may prevent metabolic disorders but are seldom effective. Hence, NAFLD is recognized increasingly as a major public health burden [24, 25]. Therefore, prevention and treatment of NAFLD are paramount for the health promotion and accordingly is focused on search for available and effective agents from natural product-derived materials safely and consistently.

In our present study, the effects of PCM on adiposity were compared with those of HFD control mice in the HFD-induced NAFLD model. In this study, the NAFLD model was successfully established after 23 weeks of HFD feeding and thereafter was treated either orlistat or PCM for 8 weeks except NAFLD control mice. This model has characteristics of excess body fat, dyslipidemia, and fatty liver [8, 26]. Namely, an elevated body weight and serum TG, TC, LDL, VLDL, and hepatic TG and TC levels in HFD control mice generally alleviated significantly by the administration of O60 and PCM. In addition, the histological alterations of livers by Oil Red O staining and morphologic alterations of adipose tissue by HE examination showed that PCM supplementation obviously reversed the increased lipid accumulation and fat size. These results suggest that PCM treatment may enhance fat utilization in the NAFLD.

Many studies have showed that AMPK is thought to play a key role in energy restriction and is proposed as a potential target for treating metabolic disorders [27]. AMPK is a sensor of cellular energy status that regulates energy balance. AMPK is a serine/threonine heterotrimeric kinase and composed of one catalytic *α*-subunit bound with *β*- and *γ*-regulatory subunits [28]. Activation of AMPK occurs with the phosphorylation of the *α*-subunit at threonine 172 (Thr172) because of the increase of the cellular AMP:ATP ratio by metabolic stresses. AMPK is also activated by several natural plant products derived from traditional medicines [29]. Previous reports showed that AMPK phosphorylation levels decreased in the liver after HFD intake [30, 31]. In the present study, we found that PCM stimulated activation of AMPK signaling pathway on HFD-induced NAFLD in mice. The net effect by activation of AMPK, a key regulator of lipid homeostasis in the liver, occurs through regulating various downstream pathways. These will increase energy production such as increase of fatty acid oxidation and diminish of glycerolipid synthesis and energy utilization [32].

The previous study proposed that PPAR*α*-mediated transcription is shown to be coactivated by the *α*-subunit of AMPK, as well as mutants of AMPK*α* such as kinase-deficient (Thr172Ala) and kinase-less (Asp157Ala, Asp139Ala). Transcriptional coactivation of PPAR*α* by AMPK*α* may complement AMPK in maintaining cellular ATP status, by linking the transcription of PPAR*α*-responsive genes such as genes coding for mitochondrial and peroxisomal *β*-oxidation of fatty acids. Moreover, AMPK*α*2 has translocated to the nucleus and induces transcription of the PPAR*α* gene [33, 34]. Thus, in the recent study, Hugan Qingzhi tablet or Monascin and ankaflavin treatment decreased lipid synthesis and increased fatty acid oxidation through activation of AMPK and PPAR*α* [35, 36]. PPAR*α* activation increases the expression of CPT-1, which is the rate-limiting enzyme of mitochondrial fatty acid oxidation, and thus facilitated lipid oxidation and decreased liver lipid deposits. Herein, we demonstrated that PCM treatment improved decreased levels of PPAR*α* and its target gene, CPT-1, in HFD control mice. Although activation of PPAR*α* showed a tendency to increase (without significance) in PCM treatment mice, CPT-1 was significantly elevated compared with HFD control mice. Furthermore, many studies reported that PPAR*α* activators upregulate UCP-2 expression in the liver. UCP-2 is mitochondrial transporters that are capable of dissipating the proton gradient and increasing thermogenesis while reducing the efficiency of ATP synthesis. UCP-2 was expressed in the liver of mice, but hepatocytes of the adult rat liver did not express UCP-2 [37]. Besides, UCP-2 plays a protective role through ameliorating oxidative stress role in palmitate-induced hepatocytic steatosis [38]. Namely, UCP-2 upregulates by either PPAR*α* or oxidative stress. Our result showed that UCP-2 significantly upregulated by O60 and PCM treatment *via* inhibition of serum and hepatic ROS as well as PPAR*α* activation.

Activation of AMPK inhibits SREBPs activity by a direct phosphorylation. SREBPs have been established as lipid synthetic transcription factors especially for cholesterol and fatty acid synthesis. SREBP-1 preferentially regulates the lipogenic process by activating genes involved in fatty acid and triglyceride synthesis, whereas SREBP-2 primarily controls cholesterol homeostasis by activating genes required for cholesterol synthesis and uptake [39, 40]. Under the normal condition, there is a low SREBP expression level in the liver, but after the adoption of HFD, there may be a significant rise in the expression of SREBPs [41]. Our results like previous study showed that phosphorylation of AMPK significantly inhibited the expression of SREBP-1 and SREBP-2 with the administration of O60 and PCM100.

Activation of SREBP-1, Ser372 phosphorylation, prevents its proteolytic processing and translocation into the nucleus and thereby promoting the lipogenic process in the liver. Then, upregulation of ACC, FAS, and SCD-1 accelerates *de novo* fatty acid synthesis [42, 43]. SREBP-2 regulates transcription of cholesterol-related genes such as HMGCR, which encodes the rate-limiting enzyme of cholesterol biosynthesis [44]. TG metabolism consists of synthesis (de novo lipogenesis) and catabolism (lipolysis and *β*-oxidation). ACC-1, FAS, and SCD-1 are key enzymes of de novo lipogenesis [45]. ACC transforms the acetyl CoA into malonyl CoA. Then, FAS synthesizes the palmitate from acetyl CoA and malonyl CoA. The palmitate is then elongated through the action of the elongase forming the stearate. Palmitate and stearate (saturated fatty acids) are subsequently desaturated on SCD1 forming palmitoleate and oleate (unsaturated fatty acids), respectively. These monounsaturated fatty acids are preferentially integrated into membrane synthesis or TG [46]. In this study, expression of lipogenic genes such as ACC, FAS, and SCD-1 in HFD control mice significantly elevated compared with normal mice. Besides, all treatment mice lowered compared with those of HFD control mice. Especially, PCM100 significantly reduced three genes. These data indicate that PCM might ameliorate HFD-induced metabolic abnormalities and also enhance the noxious condition of NAFLD.

The liver can be strongly affected by changes in the gut microbiota due to increased intestinal permeability caused by the entry of microbial antigens into the liver. Animal studies about the gut microbiota and observational studies in patients with NAFLD provided considerable evidence that dysbiosis contributes to the pathogenesis of NAFLD. Dysbiosis increases gut permeability and hepatic exposure to deleterious substances that elevate hepatic inflammation [47]. Also, recent studies revealed that the altered gut microbiota can be deeply implicated in the pathogenesis of NAFLD, hepatitis B virus (HBV), and hepatitis C virus (HCV) coinfected patients. Namely, the enhancement of the gut microbiota unbalance may be intimately related liver disease development and progression [48]. Traditional Chinese medicines (TCMs) contain phytochemical compounds, such as flavonoids, polysaccharides, alkaloids, and saponins, which are easily metabolized by the gut microbiota and directly act on the gut microbiota [49]. For example, berberine, a clinically effective drug for the treatment of NAFLD that includes isoquinoline alkaloid, has recently exerted its effects by modulating the gut microbiota [50]. Accordingly, PCM included *Diospyros kaki*, which possess microbial metabolism and could exert such action. Although not covered in this study, it is considered necessary to study additional mechanisms of PLM in NAFLD.

## 5. Conclusions

**Figure 7:**
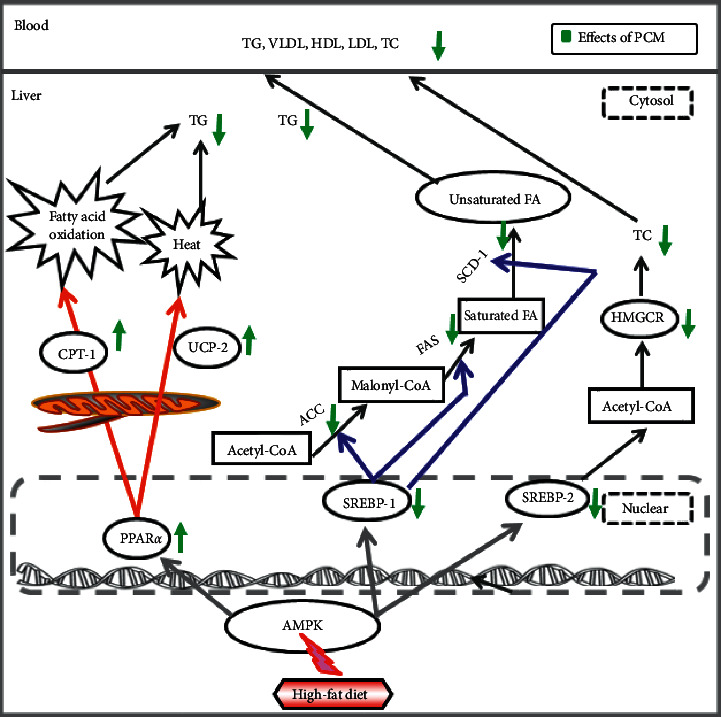
Predicted mechanism in hepatic tissues on administering PCM.

In summary, the current study suggests that PCM suppressed hepatic triglyceride accumulation through modulation of the AMPK-PPARs-SREBPs signaling pathway, as shown in Figure 7. Accordingly, PCM may be a potential therapeutic agent for treating nonalcoholic fatty liver disease.

## Figures and Tables

**Table 1:** The effect of PCM on body weight, liver weight, visceral fat weight, and food intake.

Group	Body weight (before drug treatment) Initial (g)	Body weight (drug treatment)			Liver weight (mg/g BW)	Food intake (g/day)	Calories of food intake (kcal/g)
		Initial (g)	Final (g)	Gain (g/8 weeks)			
Normal	22.06 ± 0.47	29.9 ± 0.6^*∗∗∗*^	31.8 ± 0.5^*∗∗∗*^	2.0 ± 0.3	59.6 ± 1.8^*∗∗*^	3.7 ± 0.2^*∗∗∗*^	14.2
HFD-fed mice							
Veh	22.64 ± 0.39	43.3 ± 2.2	43.8 ± 2.4	0.5 ± 0.9	45.6 ± 3.1	2.7 ± 0.2	12.8
O60	22.82 ± 0.50	43.3 ± 2.1	37.6 ± 2.9	−5.7 ± 1.0^*∗∗∗*^	35.0 ± 1.3^*∗*^	3.1 ± 1.1^*∗∗∗*^	14.7
PCM50	22.77 ± 0.75	43.3 ± 1.5	42.7 ± 1.8	−0.6 ± 0.8	42.3 ± 1.3	2.6 ± 0.1	12.3
PCM100	22.75 ± 0.55	43.3 ± 1.5	42.5 ± 1.9	−0.8 ± 0.7	33.7 ± 2.1^*∗*^	2.6 ± 0.2	12.3

Calories per gram in normal diet add to 3.85 kcal/g, and calories per g in 45%. HFD is 4.73 kcal/g. Veh, HFD control mice; O60, orlistat 60 mg/kg-treated and obese mice; PCM50, PCM 50 mg/kg-treated and obese mice; PCM100, PCM 100 mg/kg-treated and obese mice. Data are the mean ± SEM, *n* = 7. Significance: ^*∗*^*p* < 0.05, ^*∗∗*^*p* < 0.01, and ^*∗∗∗*^*p* < 0.001 versus HFD control mice.

**Table 2:** Biochemical analyses.

Parameters	Normal	HFD-fed mice			
		Veh	O60	PCM50	PCM100
*Serum*					
ROS (flu/min/mL)	5094 ± 219^*∗∗∗*^	6631 ± 941	5425 ± 294^*∗∗*^	5543 ± 180^*∗∗∗*^	5274 ± 547^*∗*^
TG (mg/dL)	187.3 ± 6.6^*∗∗*^	220.7 ± 5.7	198.9 ± 1.6^*∗∗*^	195.0 ± 2.3^*∗∗*^	189.5 ± 1.0^*∗∗∗*^
TC (mg/dL)	130.9 ± 1.4^*∗∗∗*^	196.6 ± 4.0	161.9 ± 4.6^*∗∗∗*^	184.5 ± 4.7	160.3 ± 12.2^*∗*^
HDL (mg/dL)	82.4 ± 0.5^*∗∗∗*^	96.1 ± 0.7	97.3 ± 7.1^*∗∗*^	103.8 ± 0.6^*∗∗∗*^	98.2 ± 2.7
LDL (mg/dL)	15.9 ± 1.1^*∗∗∗*^	45.1 ± 1.7	33.2 ± 2.1^*∗∗*^	39.3 ± 2.7	39.8 ± 0.9^*∗*^
VLDL (mg/dL)	39.6 ± 1.2^*∗*^	44.1 ± 1.1	39.8 ± 0.3^*∗∗*^	39.2 ± 0.6^*∗∗*^	37.9 ± 0.2^*∗∗∗*^

*Liver*					
ROS (flu/min/mg protein)	3123 ± 127^*∗*^	3870 ± 218	2507 ± 93^*∗∗∗*^	2948 ± 63^*∗∗∗*^	2630 ± 187^*∗∗*^
TBARS (nmol/mg protein)	0.12 ± 0.01^*∗*^	0.15 ± 0.01	0.11 ± 0.0^*∗∗∗*^	0.11 ± 0.01^*∗∗*^	0.11 ± 0.01^*∗∗*^
TG (mg/mg protein)	2.87 ± 0.09	3.33 ± 0.3	2.12 ± 0.06^*∗*^	2.94 ± 0.12	2.23 ± 0.05^*∗*^
TC (mg/mg protein)	2.42 ± 0.08	2.81 ± 0.25	1.76 ± 0.04^*∗∗*^	2.48 ± 0.1	2.07 ± 0.16^*∗*^

Veh, HFD control mice; O60, orlistat 60 mg/kg-treated and obese mice; PCM50, PCM 50 mg/kg-treated and obese mice; PCM100, PCM 100 mg/kg-treated and obese mice. Data are the mean ± SEM, *n* = 7. Significance: ^*∗*^*p* < 0.05, ^*∗∗*^*p* < 0.01, and ^*∗∗∗*^*p* < 0.001 versus HFD control mice.

**Table 3:** The effects of PCM on serum hepatic functional parameters.

Group	Normal	HFD-fed mice			
		Veh	O60	PCM50	PCM100
AST (IU/L)	59.23 ± 4.22^*∗∗∗*^	155.47 ± 18.93	108.80 ± 7.75^*∗∗*^	87.08 ± 4.95^*∗∗∗*^	79.86 ± 10.28^*∗∗∗*^
ALT (IU/L)	16.41 ± 1.14^*∗∗∗*^	36.38 ± 4.13	15.93 ± 1.22^*∗∗∗*^	16.73 ± 0.67^*∗∗∗*^	17.55 ± 1.12^*∗∗∗*^

Veh, HFD control mice; O60, orlistat 60 mg/kg-treated and obese mice; PCM50, PCM 50 mg/kg-treated and obese mice; PCM100, PCM 100 mg/kg-treated and obese mice. Data are the mean ± SEM, *n* = 7. Significance: ^*∗*^*p* < 0.05, ^*∗∗*^*p* < 0.01, and ^*∗∗∗*^*p* < 0.001 versus HFD control mice.

## Data Availability

The data sets used and analyzed during this study are available from the corresponding author upon reasonable request.
